# Comparative Analysis of the Thulium Fiber Laser, Holmium: YAG Laser, and Pneumatic Lithotripter in Mini-Percutaneous Nephrolithotomy: A Tertiary Care Center Experience

**DOI:** 10.7759/cureus.93209

**Published:** 2025-09-25

**Authors:** Narayan Shubham, Ujwal Kumar, Yashasvi Singh, Sameer Trivedi, Lalit Kumar, Aviral Srivastava, Sahil Data, Anil Baliyan, Madan Gopal Bhardwaj

**Affiliations:** 1 Department of Urology, Institute of Medical Sciences, Banaras Hindu University, Varanasi, IND

**Keywords:** holmium:yag laser, infundibular stenosis, mini-percutaneous nephrolithotomy, pneumatic lithotripter, stone-free rate, thulium fiber laser

## Abstract

Background

Mini-percutaneous nephrolithotomy (mini-PCNL) has become a standard treatment for moderate-sized renal calculi. Among intracorporeal lithotripters, the thulium fiber laser (TFL), Holmium: YAG laser, and pneumatic devices remain the most widely used, yet direct comparative evidence in mini-PCNL remains limited.

Aim

The aim of this study is to compare the intraoperative efficiency, perioperative outcomes, and long-term sequelae of TFL, Holmium: YAG, and pneumatic lithotripters in mini-PCNL.

Materials and methods

A comparative study was conducted in 221 patients undergoing mini-PCNL for 10-30 mm renal stones. Patients were allocated to TFL (n=79), Holmium: YAG (n=72), or pneumatic lithotripter (n=70) groups. Demographics, stone characteristics, intraoperative parameters, perioperative complications, and 12-month outcomes were analyzed. Statistical comparisons included ANOVA and chi-square tests, with significance set at p<0.05.

Results

Baseline characteristics were comparable across groups. Stone disintegration time was significantly shorter in the TFL group (24.17 ± 4.7 min) than Holmium (31.45 ± 6.3) and pneumatic groups (35.26 ± 5.9) (p<0.01). Mean operative time was lowest with TFL (53 ± 9.8 min) versus Holmium (71 ± 14.6) and pneumatic (83 ± 12.8) (p<0.001) groups. In laser groups, energy usage per case was markedly lower with TFL (15.95 kJ) compared to Holmium (23.59 kJ) (p<0.01). Stone-free rate (SFR) was highest with TFL (94.8%), followed by Holmium (84%) and pneumatic (79%) (p=0.02) groups. TFL also showed the lowest hemoglobin drop (0.44 g/dL) and hematocrit change (3.2%, both p<0.05). Complication rates by Clavien-Dindo classification were similar, though hematuria and sepsis were more frequent in the pneumatic group. At 12 months, infundibular stenosis was detected in six TFL, four Holmium, and two pneumatic patients (p=0.18). Kaplan-Meier analysis demonstrated a nonsignificant trend toward higher stenosis with TFL.

Conclusion

TFL offers superior intraoperative efficiency, reduced energy usage, and a higher SFR in mini-PCNL. However, the potential for increased long-term stenosis requires further evaluation. Clinicians should weigh immediate benefits against possible delayed sequelae when selecting the lithotripter modality.

## Introduction

Nephrolithiasis is among the most prevalent urological disorders globally. Reported lifetime prevalence rates are 7-13% in North America and 5-9% across Europe and Asia [1]. Its burden is rising, mainly due to lifestyle-related factors such as dietary changes, physical inactivity, increasing obesity, and even environmental influences like climate [2]. Recurrence is frequent (50% in five years; EAU 2022), and many patients eventually progress to surgical intervention when conservative strategies prove inadequate.

Before the advent of minimally invasive surgery, open stone surgery was the mainstay of treatment for large renal calculi, though it carried considerable morbidity, prolonged hospitalization, and delayed recovery. The introduction of percutaneous nephrolithotomy (PCNL) in the 1970s transformed renal stone management by offering high clearance with lower morbidity [3]. Since then, advances in technique and instrument downsizing have produced multiple variants, including standard, mini-, ultra-mini, and micro-PCNL, each balancing stone clearance against invasiveness [4,5].

Mini-PCNL, typically defined by tract sizes of 14-20 Fr, has gained wide acceptance for the treatment of stones between 10 and 30 mm [6]. It offers an intermediate option between retrograde intrarenal surgery (RIRS) and conventional PCNL. Compared with RIRS, mini-PCNL achieves higher stone-free rates with fewer reinterventions [6,7], while miniaturization relative to standard PCNL reduces bleeding risk, postoperative pain, and hospitalization [8]. Current guidelines therefore endorse mini-PCNL as an important frontline option for moderate stone burdens [7].

Alongside tract size, the type of intracorporeal lithotripter plays a central role in operative efficiency. Over the years, three main technologies have been widely applied: pneumatic devices, Holmium: YAG laser, and, more recently, the thulium fiber laser (TFL).

Pneumatic lithotripters were the earliest tools available, fragmenting calculi through ballistic impulses [9]. They remain simple and inexpensive, but their limitations include high retropulsion and inability to achieve effective dusting, often necessitating auxiliary procedures [10].

The Holmium: YAG laser, introduced in the 1990s, quickly became the workhorse of endourology [11]. Its wavelength (2100 nm) is strongly absorbed in water, making it effective against all stone types. It allows both fragmentation and dusting but is constrained by relatively thick fibers (200-550 µm) and limited maximum frequencies, while retropulsion remains an issue at higher pulse energies [12].

The TFL represents the latest development in intracorporeal lithotripsy. Operating at 1940 nm, with nearly ten-fold higher water absorption than Holmium, it delivers energy more efficiently to the stone-fluid interface [13]. TFL can be used with very thin fibers (down to 150 µm), supports very high pulse frequencies, and works effectively at low energies. These characteristics allow smooth dusting with minimal retropulsion [14]. In bench experiments, TFL produced ablation rates up to four times higher than Holmium and generated finer dust particles [15]. Early clinical series, including that of Enikeev et al., have confirmed shorter stone disintegration times and higher clearance rates with TFL compared to Holmium during PCNL [14]. Kronenberg and Traxer and Keller et al. likewise emphasized its efficiency and safety advantages [15,16].

Despite encouraging data, most available studies are limited in design, involve small numbers, or focus on ureteroscopy rather than percutaneous approaches [17-19]. In addition, long-term outcomes such as infundibular stenosis remain poorly characterized.

Given this context, we undertook a prospective head-to-head comparison of TFL, Holmium: YAG, and pneumatic lithotripters in mini-PCNL for stones sized 10-30 mm. Our study specifically assessed intraoperative efficiency, perioperative morbidity, stone-free rates (SFRs), and long-term complications, thereby aiming to generate clinically relevant evidence for guiding practice and future recommendations.

## Materials and methods

Study design and ethical approval

**Figure 1:**
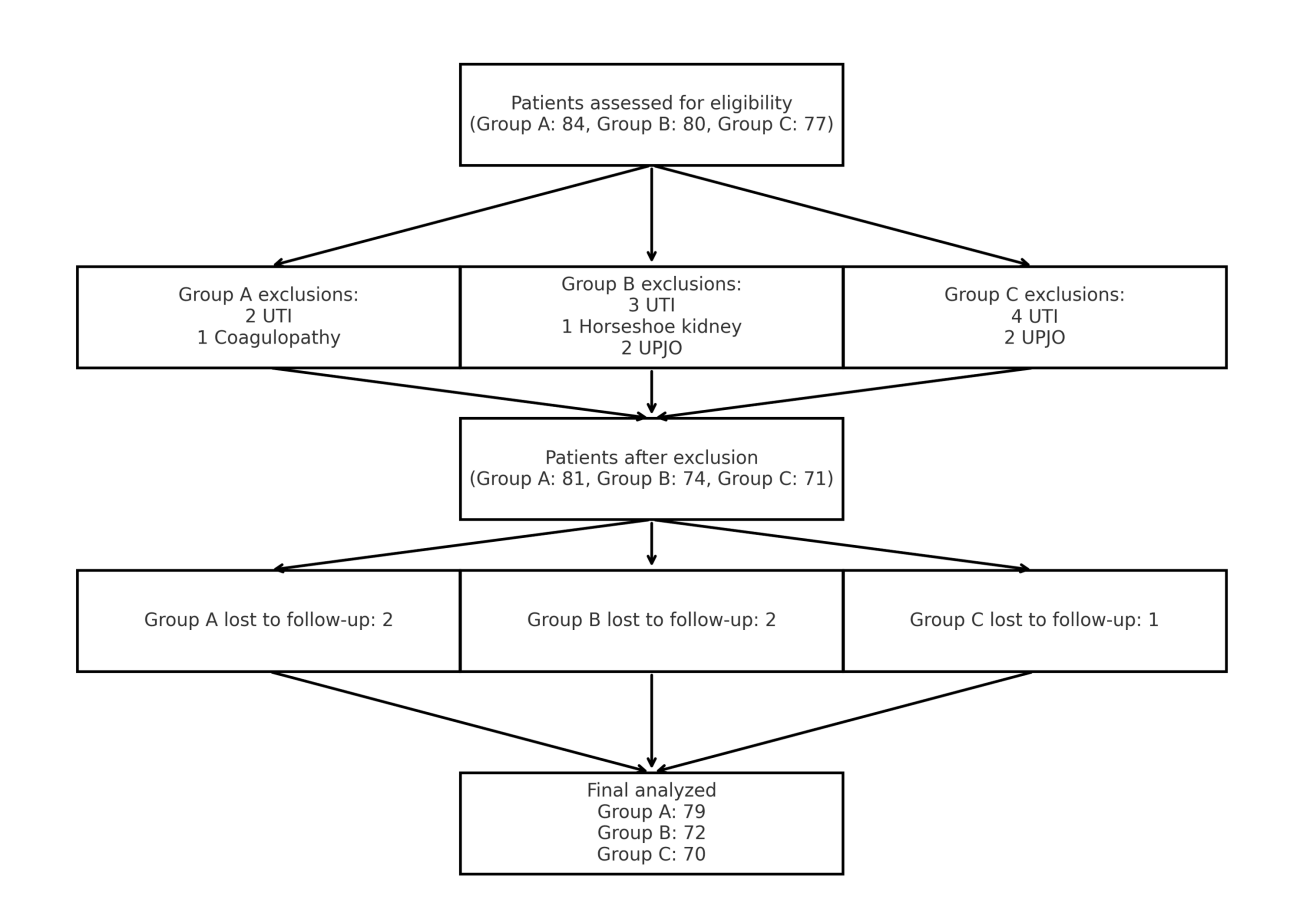
A CONSORT-style flow diagram of our study CONSORT: Consolidated Standards of Reporting Trials

This was a comparative study conducted between January 2023 and December 2024 in the Department of Urology, IMS-BHU. The study was approved by the institutional ethics committee (Ref no: Dean/ 2023/ EC/ 6803). A total of 221 consecutive patients with renal calculi measuring 10-30 mm were included. Patients were stratified into three groups according to the lithotripsy modality used during mini-PCNL: Group A (TFL, n=79), Group B (Holmium: YAG, n=72), and Group C (Pneumatic, n=70) (Figure 1). The study was designed as a prospective, three-arm comparison. We planned to detect a clinically meaningful difference in operative time between groups, based on published differences of 15-25 minutes (SD 12-15) for mini-PCNL using different lithotripters, corresponding to a large effect size (Cohen’s f ≈ 0.8-1.0). Under a one-way fixed-effects ANOVA (α=0.05, power=0.80, three groups), this effect size requires ≈12-15 patients per group (G*Power 3.1; Faul et al.) [20]. Our enrolled cohort (n=221; 79/72/70 per arm) exceeds this by >4-fold, providing >0.90 power to detect the observed between-group differences in disintegration and operative times. For the SFR, assuming absolute differences of 10-15% between modalities, our total sample also affords >0.80 power (χ² test of homogeneity, α=0.05). 

Inclusion criteria

Patients aged 18-70 years with solitary or multiple renal stones measuring 10-30 mm on CT urography were eligible. Only patients with Guy’s stone score grades I-III were included, ensuring comparable anatomical complexity. Preoperative urine culture had to be sterile, and routine hematological and biochemical investigations had to be within normal ranges.

Exclusion criteria

Exclusion criteria were as follows: active urinary tract infection, coagulopathy uncorrected at the time of surgery, pregnancy, anatomical abnormalities such as horseshoe kidney or ureteropelvic junction obstruction, prior ipsilateral open or endoscopic renal surgery, to minimize anatomical variability.

Randomization and blinding

The randomization sequence was generated using a computer-based random number generator (Microsoft Excel RAND function). The group assignments were placed in sequentially numbered, opaque, sealed envelopes prepared by a research assistant who was not involved in patient recruitment or outcome assessment. At the time of enrollment, the treating surgeon opened the next envelope in sequence to determine group allocation. While the intended allocation was equal across the three groups, minor imbalances in the final numbers (79, 72, and 70) occurred due to postoperative exclusions and loss to follow-up. Patients were assigned to Group A: TFL lithotripsy, Group B: Holmium: YAG laser lithotripsy, and Group C: Pneumatic lithotripsy. Due to the nature of interventions, surgeons could not be blinded; however, radiologists assessing stone clearance and statisticians analyzing data were blinded to group assignment. Hence, it was a single blinded study.

Preoperative evaluation

All patients underwent baseline hematology, coagulation profile, renal function tests, urine culture, USG0 KUB and CT urography for stone size, density, and volume calculation. Stone volume was calculated using the ellipsoid formula (length × width × height × π/6). Guy’s stone score [21] was assigned by two independent urologists, with discrepancies resolved by consensus. 

Surgical technique

All procedures were performed under general anesthesia with the patient in the prone position. After cystoscopy, a 5F ureteric catheter was placed in the ipsilateral ureter for retrograde pyelography. For access, fluoroscopy-guided puncture into the desired calyx was performed using an 18G needle. A 0.035-inch guidewire was advanced into the collecting system, followed by tract dilation with a single-step metallic dilator to 16-17.5 Fr. A mini-nephroscope (14 Fr) was used in all cases. Continuous irrigation (gravity dependent) was maintained at 60-80 cm H₂O pressure. The intrapelvic pressures were maintained in a low range to avoid sepsis.

Lithotripsy settings

**Figure 2:**
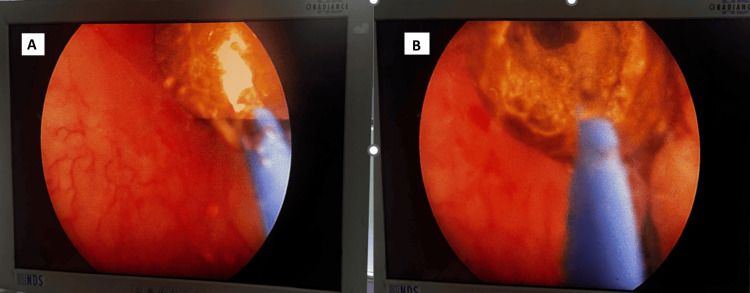
Intraoperative image of thulium fiber lithotripsy

The groups were as follows: Group A (TFL): 60W Quanta system, 400 µm fiber; pulse energy 1-1.5 J; frequency 5-18 Hz; average power 7-15 W (Figure 2), Group B (Holmium: YAG): 60W Quanta system, 550 µm fiber; pulse energy 0.8-1.2 J; frequency 8-15 Hz; average power 7-18 W, Group C (Pneumatic): Pulse pneumatic lithotripter with 72.5 psi driving pressure. Dusting settings were not used in thulium fiber laser. Only high-energy fragmentation settings were used. The settings were standardized for all the operators and the fiber used was reusable. The tips of laser fibers were shaved whenever necessary. Stone fragmentation was continued until residual fragments were <2 mm. Fragments were evacuated with forceps or flushed out by irrigation. Nephrostomy (14 Fr) was placed selectively in cases of bleeding, PCS injury, or residual fragments.

Intraoperative parameters

Intraoperative parameters include stone disintegration time, defined as the duration from the initiation of lithotripsy until complete clearance of visible stones, operative time, measured from puncture to nephrostomy placement, total energy delivered, recorded for laser groups in kilojoules, and pelvicalyceal injury, documented as perforation or mucosal tear identified intraoperatively.

Postoperative care

Hemoglobin and hematocrit were measured at 24 hours. Analgesia was standardized with intravenous paracetamol and tramadol. Antibiotics were continued for 48 hours. Uncomplicated cases were discharged on POD 3. If a nephrostomy tube was placed, it was removed on POD 2; the per urethral catheter was removed on POD 3. DJ stent was removed at six weeks. Patients were reviewed at three and six months with X-ray KUB, urine routine microscopy, and urine culture. SFR at one month was assessed by USG, with CT urography when indicated, and defined as the absence of fragments >2 mm according to guideline-based criteria. At 12 months, CT urography was performed for patients with normal creatinine to evaluate for infundibular stenosis; NCCT-KUB was used for those with deranged renal function.

Stone-free status

**Figure 3:**
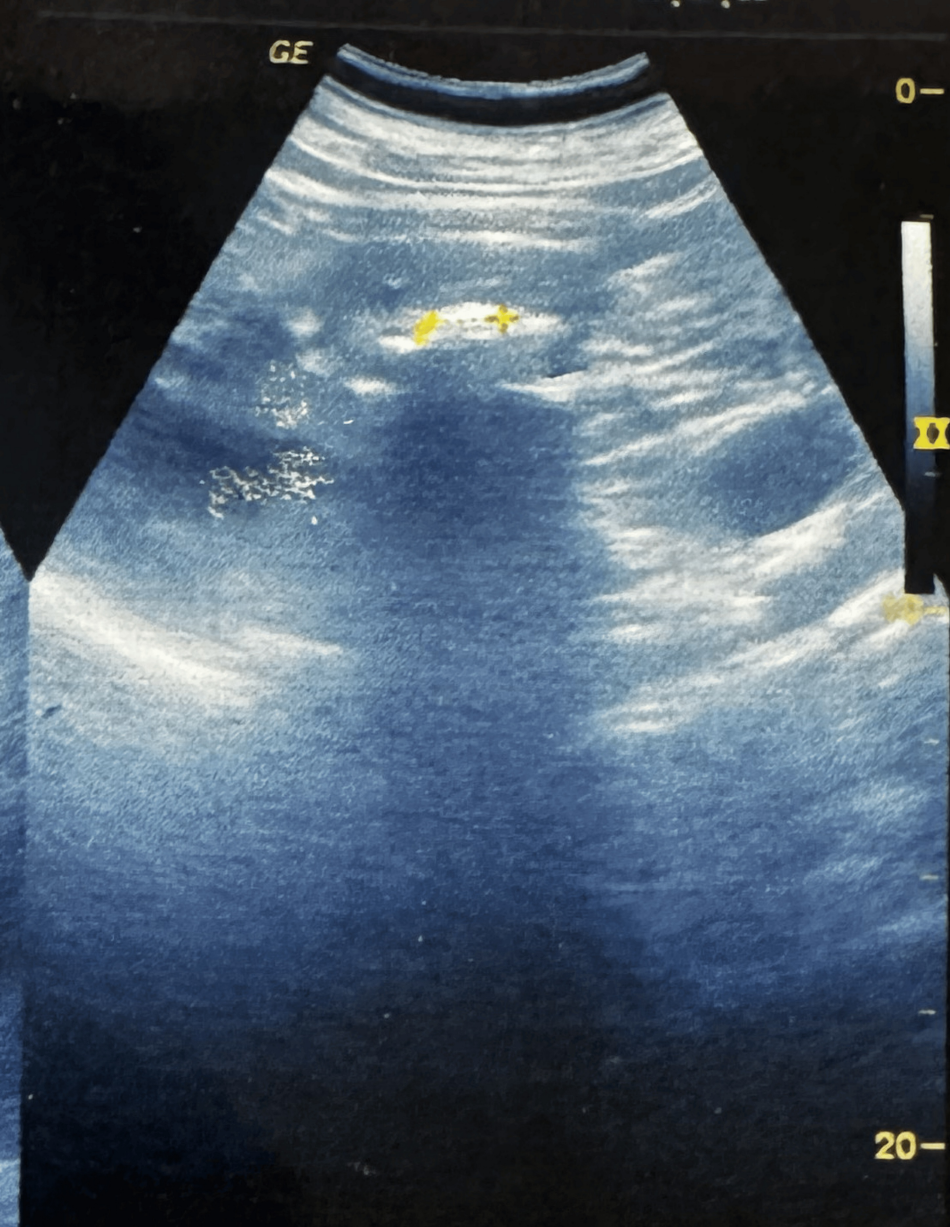
Preoperative USG KUB showing a right renal calculus as marked in the figure with a radio opaque shadow

**Figure 4:**
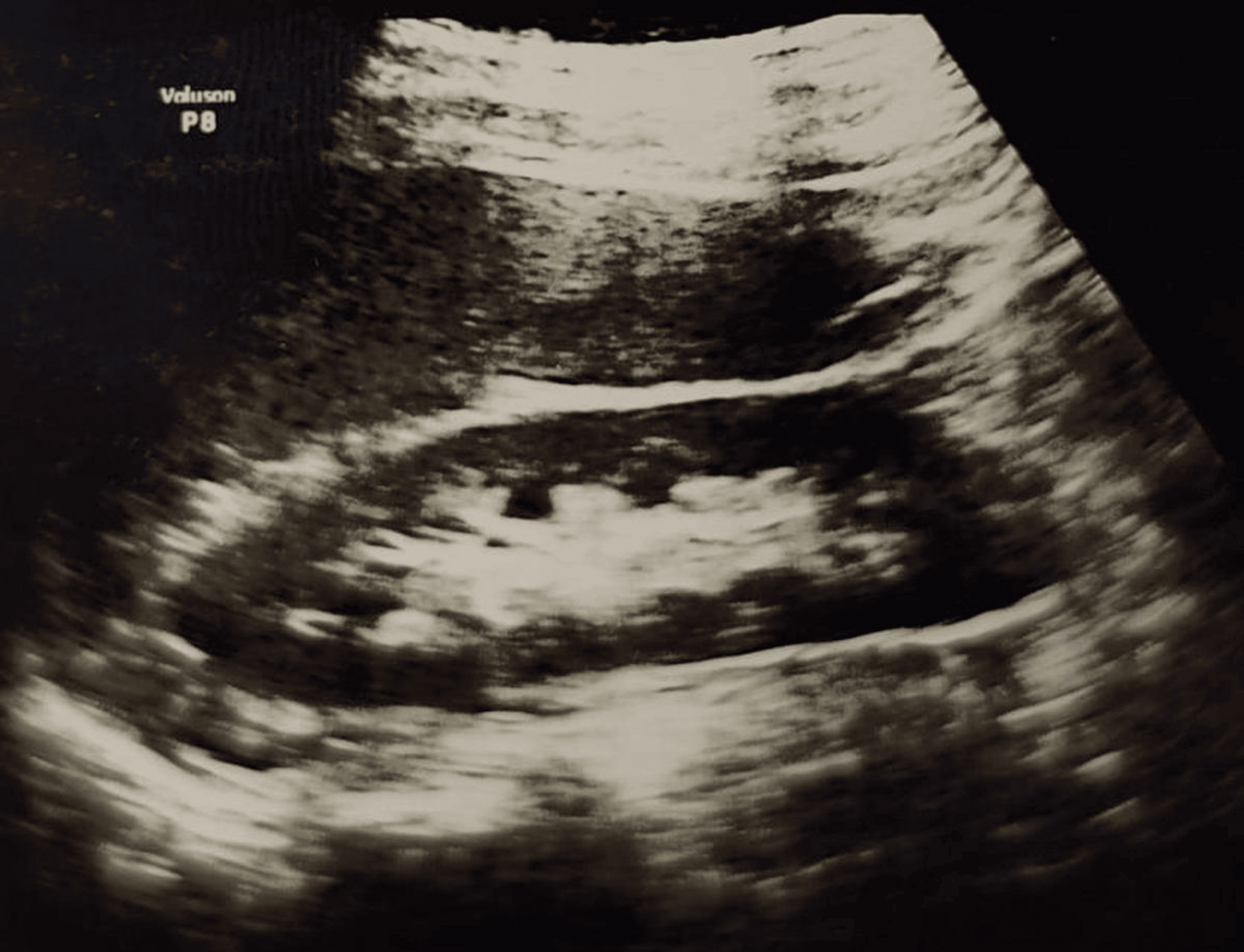
Postoperative USG KUB showing the right kidney free of stone burden

The SFR was assessed at one month using ultrasonography and CT urography when indicated. SFR was defined as absence of fragments >2 mm [5,22]. Preoperative and postoperative USG KUB of a patient who underwent right thulium laser mini-PCNL is shown in Figure 3 and Figure 4, respectively.

Complications

Intraoperative events (e.g., pelvicalyceal system injury) and postoperative complications were prospectively recorded and classified according to the Clavien-Dindo system [23]. Infectious complications (SIRS, sepsis) were defined according to standard criteria. Complications like hematuria, fever, and sepsis were specifically documented up to 30 days postoperatively. Hospital stay was calculated from the day of surgery to discharge and long-term sequelae such as infundibular stenosis were assessed at 12 months.

Follow-up protocol

Patients were followed at 3, 6, and 12 months with ultrasonography and clinical evaluation. Infundibular stenosis was diagnosed based on imaging showing calyceal narrowing with upstream dilatation, confirmed by diagnostic ureteroscopy where necessary.

Statistical analysis

Normality of continuous variables was assessed using the Shapiro-Wilk test and inspection of Q-Q plots. Homogeneity of variances was verified with Levene’s test. Where assumptions of normality and homogeneity were satisfied, comparisons were performed using one-way ANOVA with Tukey’s post-hoc tests. In case of violations, non-parametric tests (Kruskal-Wallis with Dunn-Bonferroni pairwise comparisons) were applied. Categorical variables were analyzed with χ² or Fisher’s exact tests as appropriate. A two-tailed p-value <0.05 was considered statistically significant. No interim analyses or formal stopping boundaries were planned, given the short accrual period, minimal risk profile, and the observational comparative nature of the trial. Data were analyzed using IBM SPSS Statistics for Windows, Version 26 (Released 2019; IBM Corp., Armonk, New York, United States). Continuous variables were expressed as mean ± standard deviation (SD). ANOVA was used for intergroup comparisons. Chi-square test or Fisher’s exact test was applied to categorical variables. Kaplan-Meier survival analysis was used for time-to-stenosis data, and survival curves were compared using the log-rank test. A p-value <0.05 was considered statistically significant.

Cost analysis

There was no cost involved in performing the study as our institution comes under The Central Government of India. All procedures were performed free of cost.

## Results

Baseline characteristics

A total of 221 patients were included in the final analysis: 79 in Group A (TFL), 72 in Group B (Holmium: YAG), and 70 in Group C (pneumatic). The three groups were comparable in baseline demographics (Table 1). Comparability ensured fairness in subsequent outcome analysis.

**Table 1:** Baseline characteristics Guy's stone score with [21] * signifies significant p-values i.e. < 0.05

Parameter	Group A (TFL)	Group B (Holmium)	Group C (Pneumatic)	Test statistic	p-value
Number of patients	79	72	70	—	—
Age (years, mean ± SD)	42.67 ± 15.9	42.13 ± 10.3	43.16 ± 12.3	ANOVA F(2,218)=0.11	0.897
Male (%)	62.4	54.8	61.7	χ²(2, N=221)=1.16	0.560
BMI (kg/m², mean ± SD)	24.6 ± 2.6	25.2 ± 3.16	23.8 ± 2.76	ANOVA F(2,218)=4.33	0.014*
Stone size (mm, mean ± SD)	16.8 ± 4.1	15.7 ± 2.3	16.4 ± 3.1	ANOVA F(2,218)=2.15	0.119
Stone volume (mm³, mean ± SD)	3498 ± 348	3580 ± 416	3624 ± 501	ANOVA F(2,218)=1.72	0.182
Stone size range (n)					
10–<15 mm	23	39	26		
15–<20 mm	20	21	24		
20–30 mm	36	12	20	χ²(4, N=221)=17.53	0.002*
Guy’s stone score (n) [21]					
Grade 1	45	48	34		
Grade 2	24	16	28		
Grade 3	15	8	8	χ²(4, N=221)=7.74	0.102

Intraoperative outcomes

**Figure 5:**
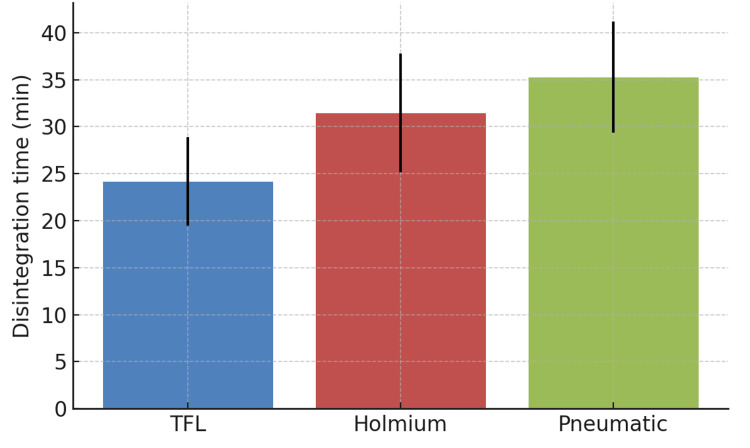
Stone disintegration time TFL: Thulium fiber laser

Stone disintegration time was significantly shorter with TFL (24.17 ± 4.7 min) than with Holmium (31.45 ± 6.3) and pneumatic (35.26 ± 5.9) (p<0.01; Figure 5). Similarly, the mean operative time was lowest in the TFL group (53 ± 9.8 min) compared to Holmium (71 ± 14.6 min) and pneumatic (83 ± 12.8 min) (p<0.001) groups. 

**Figure 6:**
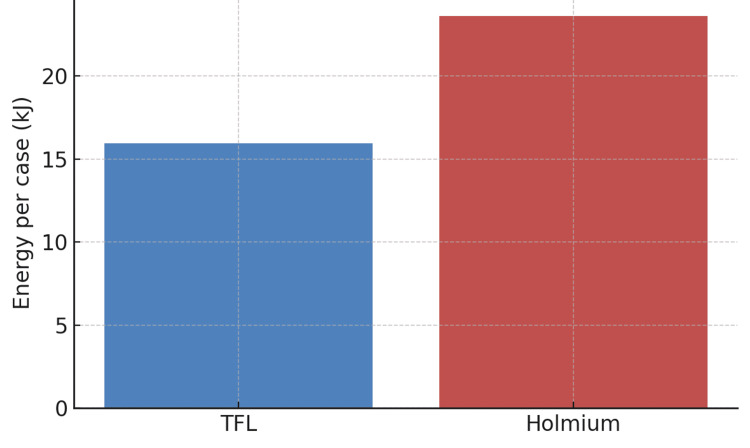
Energy used per case (lasers only) TFL: Thulium fiber laser

Among the two laser groups, energy use per case was markedly lower with TFL (15.95 kJ, 95% CI 15.34-16.57) compared to Holmium (23.59 kJ, 95% CI 22.48-24.69) (p<0.01; Figure 6). 

Pelvicalyceal system injury occurred in three patients in the TFL group, five in the Holmium group, and 11 in the pneumatic group (p=0.04). A full summary is shown in Table 2.

**Table 2:** Intraoperative findings * signifies significant p-values i.e. < 0.05 TFL: Thulium fiber laser

Parameter	Group A (TFL)	Group B (Holmium)	Group C (Pneumatic)	Test statistic	p-value
Tract size (Fr)	16–17.5	16–17.5	16–17.5	—	—
Disintegration time (min, ±SD)	24.17 ± 4.7	31.45 ± 6.3	35.26 ± 5.9	ANOVA F(2,218)=74.93	<0.001*
Operative time (min, ±SD)	53 ± 9.8	71 ± 14.6	83 ± 12.8	ANOVA F(2,218)=109.68	<0.001*
Total energy delivered (kJ, ±SD)	1260.2 ± 413.2	1698.3 ± 613.7	NA	ANOVA F(1,149)=22.6	<0.001*
Energy per case (kJ, 95% CI)	15.95 (15.3–16.6)	23.59 (22.5–24.7)	NA	ANOVA F(1,149)=28.9	<0.001*
PCS injury (n)	3	5	11	χ²(2, N=221)=7.08	0.029*

Postoperative outcomes

**Figure 7:**
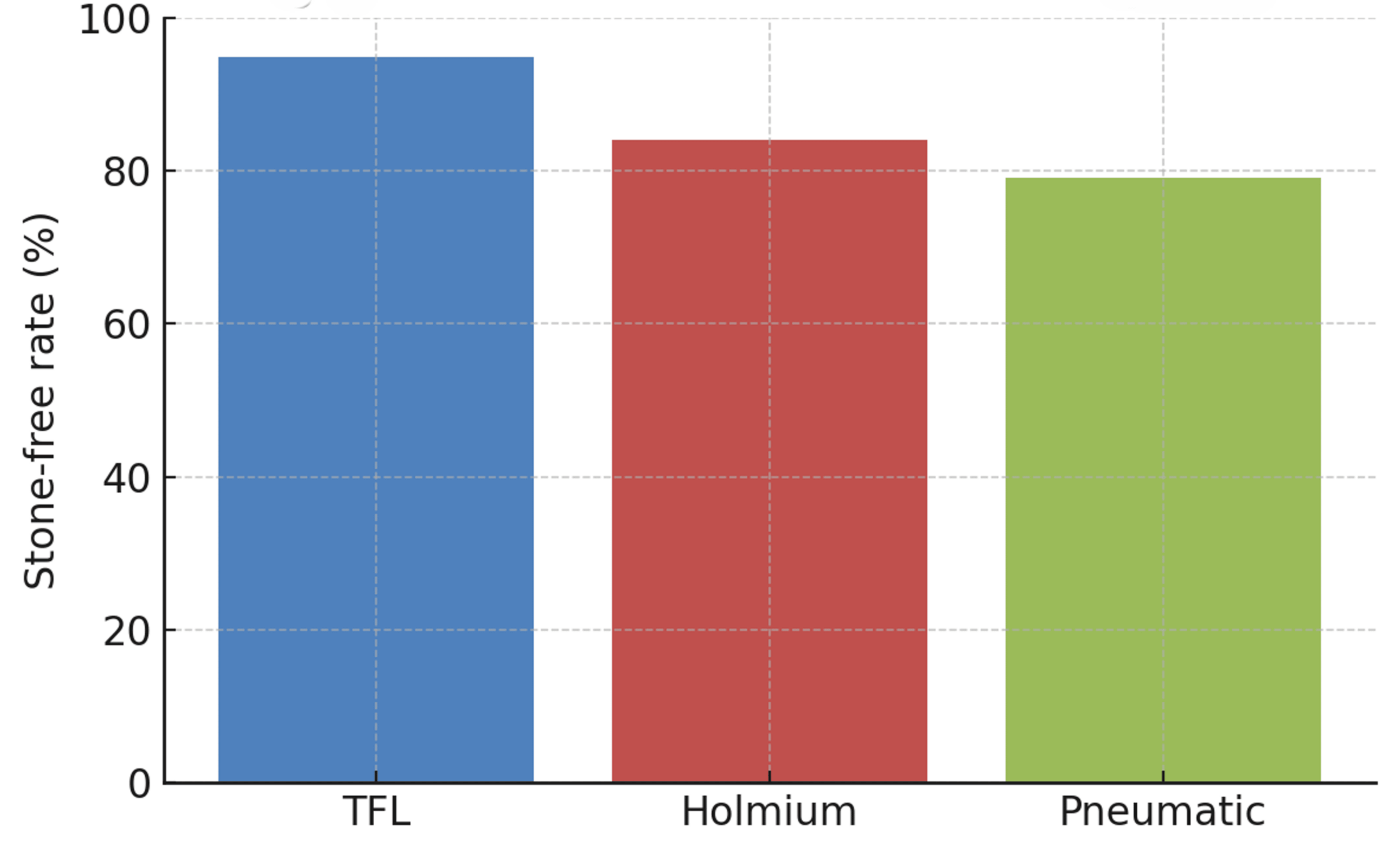
Stone-free rate across groups TFL: Thulium fiber laser

The mean hemoglobin drop was lowest in TFL (0.44 ± 0.15 g/dL) compared to Holmium (0.90 ± 0.27) and pneumatic (1.0 ± 0.3) (p<0.01). Similarly, hematocrit change was significantly smaller in TFL (3.2 ± 1.9%) compared with Holmium (4.7 ± 2.2%) and Pneumatic (5.4 ± 2.5%) (p=0.02). The SFR was highest with TFL (94.8%), followed by Holmium (84%) and pneumatic (79%) (p=0.02; Figure 7). 

Hematuria was observed in 4, 7, and 13 patients, respectively (p=0.04), while sepsis occurred in 1, 5, and 8 cases (p=0.02). Complications graded by Clavien-Dindo showed: Grade I: 6, 9, and 12 patients (p=0.12), Grade II: 3, 5, and 5 (p=0.44), Grade III: 2, 3, and 6 (p=0.21). No Grade IV or V events were recorded. Hospital stay >3 days occurred in 22.8% of TFL cases, 30.6% of Holmium cases, and 37.1% of pneumatic cases (p=0.04). These findings are summarized in Table 3.

**Table 3:** Postoperative outcomes Clavien-Dindo classification [23] TFL: Thulium fiber laser * signifies significant p-values i.e. < 0.05

Parameter	Group A (TFL)	Group B (Holmium)	Group C (Pneumatic)	Test statistic	p-value
Hb drop (g/dL, ±SD)	0.44 ± 0.15	0.90 ± 0.27	1.0 ± 0.3	ANOVA F(2,218)=112.15	<0.001*
Hct change (%, ±SD)	3.2 ± 1.9	4.7 ± 2.2	5.4 ± 2.5	ANOVA F(2,218)=19.62	<0.001*
Stone-free rate (%)	94.8	84	79	χ²(2, N=221)=7.25	0.027*
Hematuria (n)	4	7	13	χ²(2, N=221)=7.14	0.028*
Sepsis (n)	1	5	8	χ²(2, N=221)=6.53	0.038*
Clavien–Dindo classification [23]					
Grade I (n)	6	9	12	χ²(2, N=221)=3.16	0.206
Grade II (n)	3	5	5	χ²(2, N=221)=0.97	0.616
Grade III (n)	2	3	6	χ²(2, N=221)=3.01	0.222
Hospital stay >3 days (n)	18	22	26	χ²(2, N=221)=3.68	0.159
Infundibular stenosis at 12 mo (n)	6	4	2	χ²(2, N=221)=1.63	0.444

Long-term outcomes

**Figure 8:**
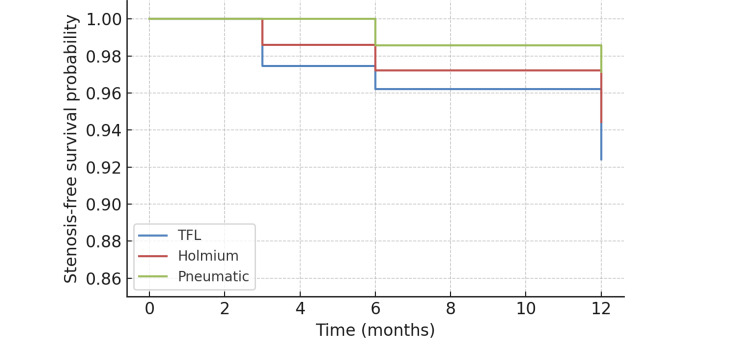
Kaplan-Meier curve showing infundibular stenosis free survival across groups TFL: Thulium fiber laser

At 12 months, infundibular stenosis was noted in six patients (7.6%) in Group A (TFL), four patients (5.6%) in Group B (Holmium), and two patients (2.9%) in Group C (Pneumatic) (p=0.18). Kaplan-Meier analysis (Figure 8) showed earlier and more frequent events in the TFL group, although overall differences across groups did not achieve statistical significance. 

**Figure 9:**
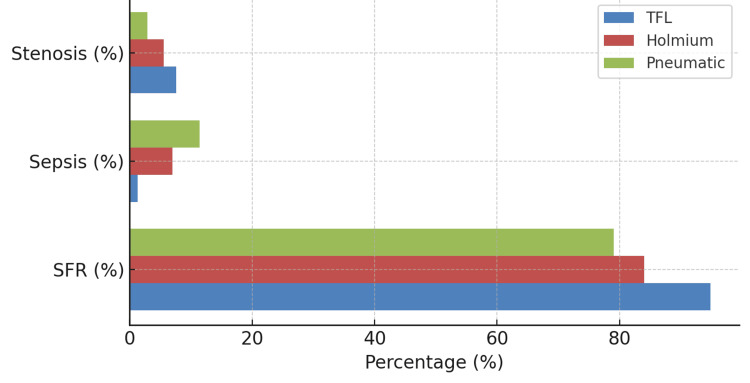
Summary of key outcomes SFR: Stone-free rate; TFL: thulium fiber laser

A visual comparison of key outcomes (SFR, sepsis, stenosis) is presented in Figure 9.

## Discussion

Mini-PCNL has become an important alternative to both standard PCNL and RIRS for moderate stone burdens. By reducing tract size, mini-PCNL lowers morbidity without compromising SFRs [6-8]. Our study is one of the few prospective comparisons of three different lithotripsy modalities, TFL, Holmium: YAG, and pneumatic devices, applied under uniform mini-PCNL conditions.

We demonstrated that TFL provided the shortest stone disintegration time (24.2 min) and lowest operative time (53 min), significantly outperforming Holmium and Pneumatic. These results parallel the findings of Enikeev et al., who in a prospective trial showed that TFL achieved faster disintegration and shorter operative duration compared with Holmium during PCNL [14]. Similarly, Kronenberg and Traxer and Keller et al. highlighted that TFL permits higher frequency and finer dusting, reducing operative times in their initial clinical experience [15,16]. In contrast, pneumatic devices, as shown in earlier literature [9,10], are limited by retropulsion and inability to dust, which prolongs operative time and necessitates fragment retrieval, an observation consistent with our findings.

In this study, we found TFL to be significantly more energy-efficient (15.95 kJ per case) than Holmium (23.59 kJ). This aligns with experimental studies demonstrating that TFL achieves higher ablation rates at lower energy settings due to its higher water absorption and thinner fibres [13]. Kronenberg and Traxer and Keller et al. similarly noted reduced energy expenditure with TFL compared to Holmium in both bench and clinical settings [15,16].

We found the highest SFR with TFL (94.8%), compared to Holmium (84%) and pneumatic (79%). Enikeev et al. also reported superior SFR with TFL over Holmium in PCNL [14]. Mahajan et al. compared laser-based PCNL to pneumatic devices and found higher clearance rates with lasers [17], a pattern reproduced in our series. Thus, the superiority of laser lithotripsy-particularly TFL, over pneumatic devices for achieving complete clearance is consistently supported by prior evidence.

The TFL group showed the lowest hemoglobin drop and hematocrit change, consistent with the safety advantages attributed to miniaturized tracts [7,8]. Complications were generally minor and evenly distributed, although hematuria and sepsis were more frequent in the Pneumatic group. This is in line with previous observations that longer operative times and greater retropulsion may increase complication risk with pneumatic lithotripters [10]. The absence of Grade IV-V complications reflects the overall safety of mini-PCNL across all modalities, as highlighted in a prior systematic review [19].

A distinctive aspect of our study was the 12-month follow-up for infundibular stenosis, which revealed a higher incidence with TFL (7.6%) than Holmium (5.6%) and pneumatic (2.9%) groups. While the difference was not statistically significant, this trend has not been widely reported in the literature. Most prior studies, such as those by Enikeev [14] and Kronenberg [15], focused on intraoperative efficiency and short-term safety, without extended follow-up for calyceal complications. Our data raises the possibility that thermal injury during TFL use may contribute to delayed stenosis, particularly when higher energy is delivered in confined calyces. This observation warrants multicenter confirmation.

For mini-PCNL, TFL offers the greatest intraoperative efficiency (shortest disintegration/operative times) and highest SFR, potentially reducing theatre time and early morbidity. However, our 12-month data suggest a higher incidence of infundibular stenosis with TFL than with Holmium or pneumatic (non-significant trend). Surgeons should therefore balance short-term gains against possible late risks, especially when delivering high energy in confined calyces, consider lower-energy/high-frequency dusting settings, and ensure structured long-term follow-up in laser-treated patients. Pneumatic devices, while less efficient, may be considered in scenarios where long-term calyceal injury risk is of particular concern.

Our study has certain limitations. First, although adequately powered, it was conducted as a pragmatic study without any planned interim analyses or stopping rules, which may limit early detection of rare or emerging safety signals. Second, blinding of surgeons was inherently not feasible due to the nature of the interventions, though outcome assessors and analysts were blinded to minimize bias. Third, while the prospective design and standardized protocols strengthen internal validity, the single-center setting and real-world variability in patient selection and stone characteristics may affect external generalizability. Finally, long-term outcomes beyond 12 months were not assessed, and further multicentric studies with longer follow-up are warranted to confirm these findings.

## Conclusions

This prospective comparative study confirms that TFL is the most efficient modality for mini-PCNL, providing the shortest disintegration and operative times, lowest energy requirement, minimal blood loss, and the highest SFR compared with Holmium: YAG and pneumatic lithotripters. However, a noteworthy observation in our series was the higher incidence of infundibular stenosis, though statistically insignificant, with TFL at 12 months, a complication that has not been widely reported in earlier studies, highlighting the importance of long-term follow-up and cautious use of high-power laser settings, especially in confined calyceal spaces. In contrast, pneumatic lithotripters, though less efficient intraoperatively and associated with lower clearance rates, exhibited the lowest stenosis rate in our cohort. This suggests a potential trade-off between immediate surgical efficiency and long-term safety.

In summary, TFL appears to be the most effective lithotripsy modality for mini-PCNL in the short term, but its possible association with delayed stenosis warrants further investigation. The results of our study are preliminary, center-specific, and limited by a short follow-up. Until larger, longer, multicenter trials are available, clinicians should carefully balance the advantages of TFL with consideration for patient-specific factors and long-term outcomes.
